# The alleviating effect and mechanism of GLP-1 on ulcerative colitis

**DOI:** 10.18632/aging.204953

**Published:** 2023-08-17

**Authors:** Wenrui Wang, Chuan Zhang, Haolong Zhang, Luyao Li, Tingting Fan, Zhenjing Jin

**Affiliations:** 1 Department of Hepatopancreatobiliary Medicine, Digestive Diseases Center, The Second Hospital, Jilin University, Changchun 130000, PR China; 2 Department of Endocrinology, The Second Hospital of Jilin University, Changchun 130000, PR China; 3 Department of Gastrointestinal and Colorectal Surgery, China-Japan Union, Hospital of Jilin University, Changchun 130000, PR China

**Keywords:** GLP-1, ulcerative colitis, inflammation, intestinal barrier, gut microbiota

## Abstract

Ulcerative Colitis (UC) is a major type of chronic inflammatory bowel disease of the colonic mucosa and exhibits progressive morbidity. The incidence and prevalence of UC is increasing worldwide. The global burden of UC, which can substantially reduce quality of life, is clearly increasing. These data highlight the need for research into prevention of UC and innovations in health-care systems to manage this complex and costly disease. Glucagon-like peptide-1 (GLP-1), a new antidiabetic drug, is used to treat Type 2 Diabetes Mellitus (T2DM). Accumulating evidence suggests that GLP-1 has additional roles other than glucose-lowering effects. Despite the abundance of GLP-1 research, studies in UC have been less consistent, especially body weight; for example, body weight, colon length, colon injury score, intestinal microbiota, remain to be studied further. To date, the molecular mechanism of the protective effect of GLP-1 on UC remains obscure. The effect of GLP-1 was studied by using a dextran sulfate sodium (DSS)-induced colitic mice and lipopolysaccharide (LPS) treated RAW264.7 cells (macrophage cell line) under *in vivo* and *in vitro* conditions, respectively. Our results indicate that GLP-1 significantly relieves ulcerative colitis as it represses the production of proinflammatory mediators. In addition, GLP-1 blocks the activation of the protein kinase B (AKT)/nuclear factor-κB (NF-κB), and mitogen-activated protein kinase (MAPK) signaling pathways. GLP-1 also alleviates DSS-induced injury to the intestinal mucosa and dysbiosis of gut microbiota. Altogether, GLP-1 has protection effect on ulcerative colitis. Thus, GLP-1 can be considered as a potential therapeutic candidate for the treatment of UC.

## INTRODUCTION

Ulcerative colitis (UC) is a chronic, recurrent, and non-specific gastrointestinal inflammation disease [1, 2] which affects people of all ages. Recent epidemiological studies have shown its highest prevalence among North Americans, even though the incidence rate is quite high among Asians [3, 4]. A global rising incidence of UC is a matter of concern as it places a burden on society. UC is a complex disease characterized by abdominal cramps, diarrhea, bloody stools, and other systemic symptoms [5]. The drugs currently used for the treatment of UC have limited efficacy due to the high recurrence rates observed and various side effects [6]. It has thus become imperative to develop new drugs and therapeutics for the treatment of UC.

The pathogenic mechanisms of UC have attracted the attention of the scientific world. Apart from genetic susceptibility, factors such as inflammatory response, dysbiosis of the intestinal microbiome, and dysfunction of the intestinal barrier promote the etiopathogenesis of UC [7]. There is sufficient evidence to believe that inflammatory cytokines such as interleukin [8]-1β, IL-6, and tumor necrosis factor-α (TNF-α) participate in disease development, and blocking these cytokines can prevent tissue damage in UC [9]. The intestinal mucosal surface acts as a barrier to maintain homeostasis between immune cells and the gut microbiome [10]. In UC, a disrupted intestinal barrier leads to the translocation of the gut microbiota into the intestinal tissue resulting in inflammation [11].

The peptide hormone GLP-1, secreted by intestinal endocrine L cells, functions to regulate metabolic processes [12]. Several studies have revealed that GLP-1 is involved in pathophysiological processes that can lead to inflammation, cardiovascular diseases, and nervous system diseases [13–15]. Interestingly, GLP-1 inhibits the NF-κB signaling pathway and alleviates acute lung injury in LPS-induced mice [16]. Wang et al. showed that GLP-1 analog liraglutide reduced the TNF-α and IL-1β expression in the hippocampus of rat pups and prevented neuroinflammation [17]. Also, Arivarasu et al. have examined the effect of GLP-1 as nanomedicine and showed its efficacy in reducing intestinal mucosal inflammation [18]. Moreover, GLP-1R agonists liraglutide can improve intestinal permeability [8]. Despite the abundance of GLP*-*1 research, studies in UC have been less consistent, especially body weight; for example, body weight, colon length, colon injury score, intestinal microbiota, remain to be studied further. However, the specific role of GLP-1 in alleviating colitis in DSS-induced mice is still unclear.

The model of UC induced by DSS in mice provides data that are widely used in pharmacological studies to find better therapeutic agents to treat UC in humans [19]. Here, we have investigated a possible protective role of GLP-1 in DSS-induced mice and LPS-exposed RAW 264.7 macrophage cells.

## MATERIALS AND METHODS

### Materials

GLP-1 (Benaglutide Injection) was obtained from Shanghai Renhui Biopharmaceutical Co., Ltd. (Shanghai, China); DSS (36–50 kDa) from MP Biochemicals (Santa Ana, CA, USA); and TRIzol reagent from Invitrogen (Carlsbad, CA, USA). Primary antibodies for Occludin and ZO-1 were purchased from Abcam (Cambridge, United Kingdom); Akt, p-Akt, NF-κB p65, p-NF-κB p65, P38, p-P38, JNK, p-JNK, ERK1/2, p-ERK1/2 from Cell Signaling Technology (Danvers, MA, USA); β-actin was obtained from Bosterbio (Pleasanton, CA, USA). Antibodies for nitric oxide synthase (iNOS) and cyclooxygenase 2 (COX2) were purchased from Abcam (Cambridge, United Kingdom). ELISA kit was obtained from BioLegend (San Diego, CA, USA).

### Animal study

Animal studies with C57BL/6 male mice (7–8 weeks old;18–20 g in weight) purchased from Liaoning Changsheng Biotechnology Co. were carried out according to the institutional animal welfare guidelines of the Experimental Animal Center of Jilin University. The forty-eight experimental animals were randomly assigned to six groups (*n* = 8 per group): a no treatment (NT) group, a GLP-1 (0.4 mg/Kg) group, a DSS (2%) group, and three DSS + GLP-1 (0.1, 0.2 and 0.4 mg/Kg) groups [20–23]. The mice of NT and GLP-1 group were given sterile water, the DSS and DSS + GLP-1 groups had free access to 2% (wt/vol) DSS supplement water for seven days induced colitis model. In the DSS + GLP-1 treated group, GLP-1 was given to mice (0.1, 0.2, or 0.4 mg/Kg) once daily for 72 h before DSS treatment and continued throughout the course of the experiment. Mice in GLP-1 group were intraperitoneally injected with GLP-1 (0.4 mg/Kg) once daily during the experiment period. GLP-1 was administered throughout the experiment. We supervised and recorded the body weight, diarrhea index and rectal bleeding of the mice daily. As previously reported, disease activity index (DAI) was evaluated by weight loss, diarrhea index, and rectal bleeding detection based on a standard scoring system [21]. The clinical scoring methods are shown in Table 1.

**Table 1:** The scoring system of clinical score.

**Score**	**Body weight decrease rate**	**Fecal property**	**Hematochezia status**
0	0%	Normal	Normal
1	1–5%	Semi loose (+)	Feces with occult blood (+)
2	6–10%	Semi loose (++)	Feces with occult blood (++)
3	1–15%	Loose (+)	Bloody feces (+)
4	>15%	Loose (++)	Bloody feces (++)

### Cell culture

The RAW264.7 cells, obtained from the Cell Bank of Type Culture Collection of Chinese Academy of Sciences (Shanghai, China), were cultured in DMEM (Corning, USA) with 10% fetal bovine serum (FBS; Gibco, USA). The cultured cells were grouped as follows: NT, LPS (1 μg/mL), GLP-1 (50 μmol/L), LPS (1 μg/mL) + GLP-1 (10, 20, and 50 μmol/L) groups. RAW264.7 cells were seeded into six-well plates and incubated at 37°C with 5% CO_2_, after 24 h of adhesion, were treated with GLP-1 (10, 20, and 50 μmol/L) for 4 h and then co-exposed to LPS (1 μg/mL) for 24 h. We diluted GLP-1 with DMEM to achieve final concentrations of 10, 20, and 50 μmol/L, respectively. Cultured cells were harvested, and RNA and proteins were extracted.

### Cell viability

The measurement of viable cell mass was performed by CCK-8 (Dojindo, Japan). Cells (3 × 10^3^ cells/well) with triplicate were firstly seeded in 96-well flat-bottomed plates for 24 h. After different concentration of GLP-1 (10, 20, 50, 100, 200, 400 μmol/L) treatment for 24 h, 10 μl solution from CCK-8 was added to each well. These plates were continuously incubated for 80 min in a humidified CO_2_ incubator at 37°C. The absorbance was measured using an enzyme-linked immunosorbent assay plate reader (Bio-reader) with a reference wave length of 450 nm.

### Hematoxylin-eosin (H&E) staining

Mice were anesthetized, sacrificed, and the colorectum (from ileum-colon junction to the proximal rectum) part was collected. The researchers measured the colon lengths and sectioned the colon tissue for *ex vivo* analysis. Colonic tissue samples were collected from lesion sites (2 × 6 mm), fixed with 4% paraformaldehyde, embedded in paraffin, sliced (5 μm thick), and stained with H&E stains as per the manufacturer’s protocol [24]. Histological changes were observed under the light microscope at a magnification of 100X. Pathological changes were then evaluated independently by two trained pathologists. The evaluation criteria were as follows: (1) epithelium loss, (2) crypt damage, (3) goblet cells depletion, and (4) inflammatory cell infiltration. The remaining extracted tissues were stored in liquid nitrogen and used later for western blot and cytokine analyses [25].

### Myeloperoxidase (MPO) and ELISA tests

The colon tissue sample was weighed and immersed in the HEPES solution (0.1 g/6000 μL). The sample was homogenized (at 50 times per minute), centrifuged, and the supernatant was collected. Homogenized protein was centrifuged at 4°C for 30 min at 14000 g. The concentration of TNF-α, IL-1β, and IL-6 in the supernatant was quantified by ELISA Kit. Similarly, 0.5% cetyltrimethylammonium chloride (CTAC) was added to the supernatant to detect MPO activity as per the manufacturer’s instructions [26].

### Intestinal microflora analysis

Colon contents were collected and stored at –80°C with a minimum time delay to preserve the microorganisms. Subsequently, the genomic DNA was extracted, sequenced, and libraries constructed using the NEB Next^®^ Ultra^™^ DNA Library Prep Kit. Index codes were installed to generate indexed libraries. Sequencing services were provided by Shanghai Applied Protein Technology Co., Ltd. Sequences analyses were performed using the UPARSE clustering algorithm. Sequences with a similarity ≥ of 97% were classified under the same OTUs as per Venn diagrams. Alpha-diversity was estimated using ACE, Chao1, Simpson, and Shannon indices. Cluster analysis was followed by Principal component analysis (PCA). Finally, Principal Coordinate Analysis (PCoA) for beta diversity was estimated using the unweighted unifrac distance matrix method. We used KRONA diagrams to graphically represent the relative abundance of the species. Linear discriminant analysis Effect size (LEfSe) was utilized to determine the dissimilarity between observed and expected taxonomic abundance. COG and KEGG databases provided a systematic functional analysis of the predicted genes.

### Western blotting

Proteins were extracted from RAW264.7 cells and colon tissue sample in RIPA lysis buffer (Beyotime, China) and quantified by the BCA method. Equal amounts of protein (30 μg) were loaded onto 10% SDS-PAGE gel, then electrophoresed and transferred to 0.2 μm PVDF membrane (Millipore, Darmstadt, Germany), which were blocked with 5% BSA for 2 h and incubated with the following primary antibodies overnight at 4°C: against COX-2 (1:1000), iNOS (1:2000), p-NF-κB p65 (1:1000), NF-κB P65 (1:1000), p-AKT (1:2000), AKT (1:2000), p-P38 (1:2000), P38 (1:1000), p-ERK1/2 (1:2000), ERK1/2 (1:2000), p-JNK (1:2000), JNK (1:2000), and β-actin (1:2000). In the following steps, the membranes were washed with TBST, and rinsed five times (10 minutes each), then incubated with corresponding HRP-labeled secondary antibodies (1:3000) at room temperature for 1 h. The immunoreactive protein bands were detected following the protocol mentioned in the chemiluminescence Kit (Beyotime, China).

### Real-time PCR

Total RNA was extracted with TRIzol and reverse transcribed to cDNA as directed in the RT-PCR Kit (ThermoFisher Scientific, Waltham, MA, USA) used here. The expression level of genes was quantitated with SYBR Green Master Mix (Roche, Shanghai, China) as per the kit’s prescribed protocol. The sequences of the primers used are presented in Table 2.

**Table 2:** Primers used for real-time PCR in this study.

**Genes**	**Sequence of primers (5′→3′)**
*iNOS*	F: CACCCAGAAGAGTTACAGC
	R: GGAGGGAAGGGAGAATAG
*COX-2*	F: GGAGGGAAGGGAGAATAG
	R: CTTGTAGTAGGCTTAAACATAG
*IL-1β*	F: ACCTGTGTCTTTCCCGTGG
	R: TCATCTCGGAGCCTGTAGTG
*IL-6*	F: AGTTGTGCAATGGCAATTCTGA
	R: AGGACTCTGGCTTTGTCTTTCT
*TNF-α*	F: GCCTCCCTCTCATCAGTTCTA
	R: GGCAGCCTTGTCCCTTG
*β-actin*	F: TGCTGTCCCTGTATGCCTCT
	R: TTTGATGTCACGCACGATTT

### Immunofluorescence

The paraffin sections of colonic tissue were dewaxed and rehydrated in gradient alcohol, then the sections were deparaffinized with xylene and rehydrated, then submerged into EDTA antigenic retrieval buffer and microwaved for antigenic retrieval. Then the slides were rinsed thrice with 0.01 M PBST for 5 min each. The tissue slides were incubated with primary antibody overnight and then washed. The sections were fluorescently coupled with a specific secondary antibody (400 times diluted in PBS) and incubated for 2 hours. Slides were stained with DAPI for 10 minutes and fixed with an anti-fading reagent. Finally, the images were captured with a fluorescence microscope (Nikon, Tokyo, Japan).

### Statistical analysis

A combination of SPSS 20.0 (SPSS, USA) and GraphPad Prism 8 (GraphPad Prism, USA) was used to analyze all statistical testing. All data represent mean ± SD. We analyzed statistical differences between groups/treatments using one-way ANOVA and Tukey test. The significance of the data between the two groups was analyzed by Student’s *t*-test. The values of *p* < 0.05 and *p* < 0.01 imply a significant difference and highly significant difference, respectively.

### Availability of data and materials

The datasets used during the current study are available from the corresponding author on reasonable request.

## RESULTS

### GLP-1 alleviates DSS-induced colitis symptoms in mice

**Figure 1:**
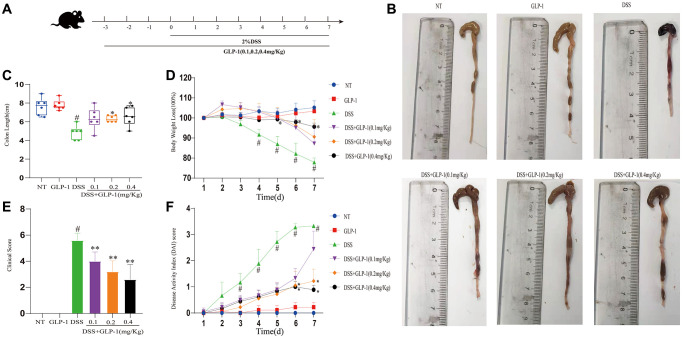
**GLP-1 alleviates DSS-induced colitis symptoms in mice.** (**A**) GLP-1 treatment on the DSS-induced colitis mouse model. (**B**) The integral colon images. (**C**) The total colon length. (**D**) Bodyweight loss. (**E**) Clinical score. (**F**) DAI score. Data are presented as mean ± SD (*n* = 6 per group). For GLP-1-DSS vs. DSS group, ^*^p < 0.05 - ^**^p < 0.01 and for DSS vs. NT group, ^#^*p* < 0.05 were considered significant.

To understand the role of GLP-1 in UC, C57BL/6 male mice were supplemented with 2% (W/V) DSS aqueous solution for seven days. These mice were intraperitoneally injected with either GLP-1 or saline (Figure 1A). Shortening of the colon is a morphological indicator of inflammatory response in DSS-induced colitic mice. As shown in Figure 1B, colon length was significantly shortened in DSS treated group as compared to the uninduced mice. After the treatment with GLP-1 shrunken colon expanded (Figure 1C). The three different concentrations of GLP-1 treatments prevented the weight loss to varying degrees during DSS exposure (Figure 1D). In Figure 1D, 1F, the DSS + GLP-1 (0.4 mg/kg) is significant with the DSS group. DAI score, including weight loss, rectal bleeding, and diarrhea, indicates the severity of the colitis disease. DSS treatment resulted in the loss of body weight with aggravated rectal bleeding and diarrhea, exhibited significantly increased clinical score and DAI score relative to control animals. GLP-1 treatment ameliorated these conditions (Figure 1E, 1F).

### GLP-1 improves DSS-induced colitis damage in mice

**Figure 2:**
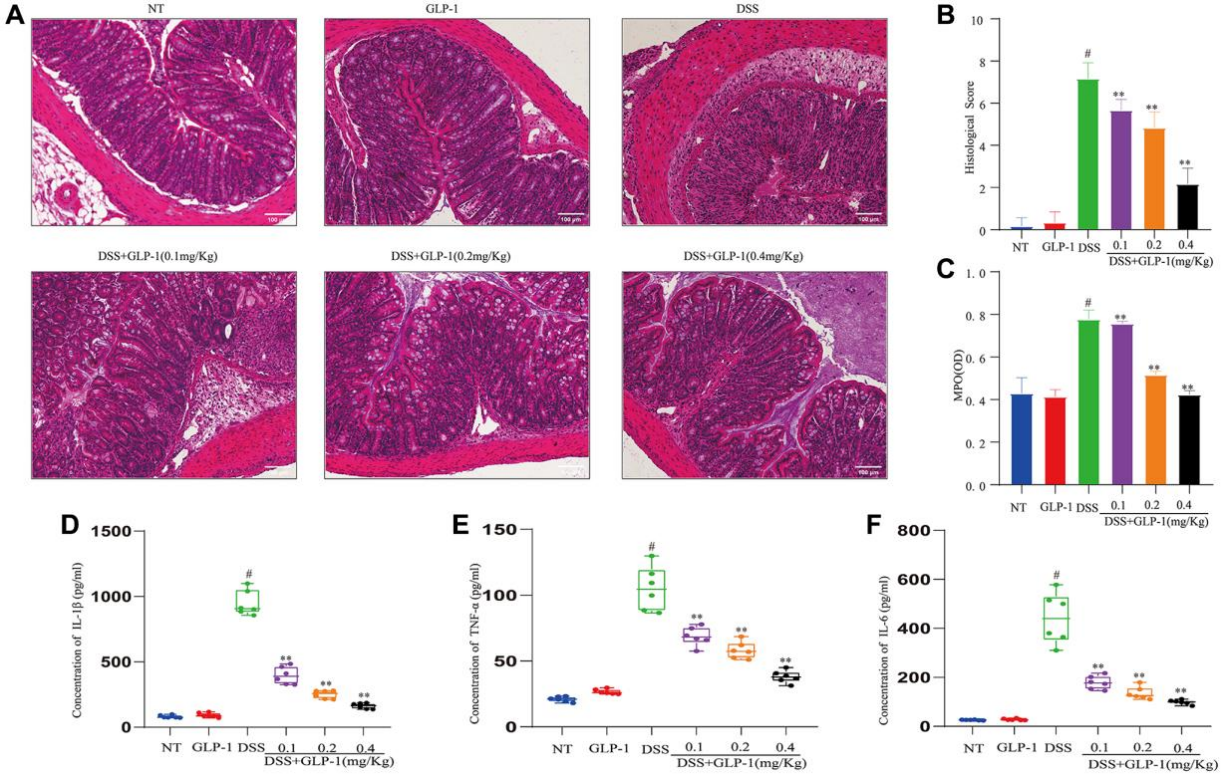
**GLP-1 improves DSS-induced colitis damage in mice.** (**A**) H&E-stained sections (100X). (**B**) Histopathological scores. (**C**) MPO activities. (**D**) The levels of IL-1β in colonic tissues. (**E**) The levels of TNF-α in colonic tissues. (**F**) The levels of IL-6 in colonic tissues. Data are presented as mean ± SD (*n* = 6 per group). For GLP-1-DSS vs. DSS group, ^*^p < 0.05 - ^**^p < 0.01 and for DSS vs. NT group, ^#^*p* < 0.05 were considered significant.

We stained tissues to detect any histological changes. We found the normal colonic architecture disruption, severe mucosal necrosis, inflammatory cell infiltration and goblet cell and crypt numbers reduction in DSS-induced mice with colitis. However, the biopsy results of the control and the GLP-1-treated mice showed normal colon tissue morphology. GLP-1 supplementation had healed the intestinal lesions (Figure 2A). Histopathological scores of mice treated with DSS alone were substantially higher than those treated with GLP-1 (Figure 2B). In addition, MPO activity (Figure 2C) and the expression of IL-1β (Figure 2D), TNF-α (Figure 2E), and IL-6 (Figure 2F) was significantly enhanced in the DSS-induced mice (*p <* 0.01); considerably abated with GLP-1 treatment. Thus, our data suggest that GLP-1 ameliorates DSS-induced intestinal inflammation and mucosal damage in mice.

### Effect of GLP-1 on the phosphorylation of Akt/ NF-κB p65 and MAPK in DSS-induced colitic mice

**Figure 3:**
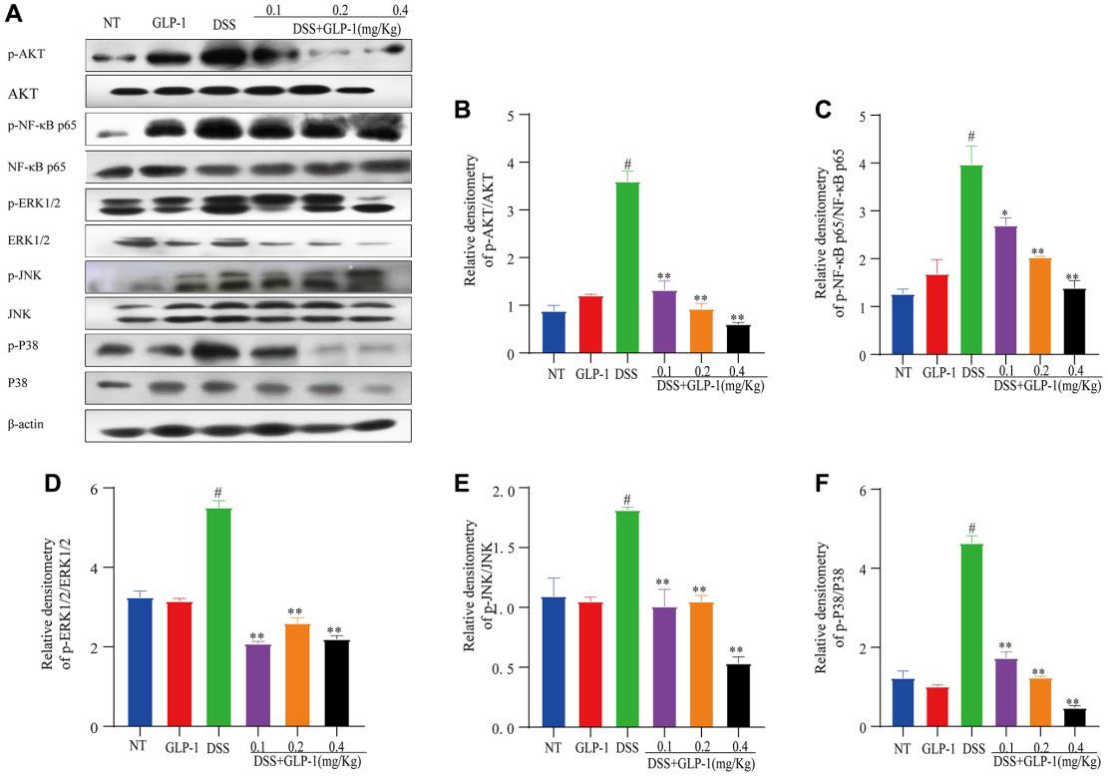
**Effect of GLP-1 on the phosphorylation of AKT/NF-κB p65 and MAPK in DSS-induced colitic mice.** The protein levels of p-AKT (**A**, **B**), p-NF-κB p65 (**A**, **C**), p-ERK1/2 (**A**, **D**), p-JNK (**A**, **E**) and p-P38 (**A**, **F**) were analyzed by western blotting. Data are presented as mean ± SD (*n* = 6 per group). For GLP-1-DSS vs. DSS group, ^*^p < 0.05 - ^**^p < 0.01 and for DSS vs. NT group, ^#^*p* < 0.05 were considered significant.

We measured the protein levels of NF-κB p65 and AKT (transcription regulator of NF-κB) through western blotting in the colitic tissues. We found that GLP-1 significantly inhibits the activation of NF-κB p65 and AKT in DSS-induced colitic mice models (Figure 3A–3C). The ERK1/2, JNK and P38 also play a significant role in the inflammatory response. Compared to the NT group, the phosphorylation expression of ERK1/2, JNK and P38 in DSS treated mice group was elevated (*p <* 0.01), this atypical rise could be reversed with the GLP-1 treatment (Figure 3A, 3D–3F). As expected, the supplement of GLP-1 reversed the DSS-induced abnormal phosphorylation of NF-κB p65, AKT, ERK1/2, JNK and P38. These data suggest that GLP-1 provides a protective effect in DSS-induced colitic mice by regulating key participants of the signaling pathways- AKT/NF-κB p65 and MAPK.

### GLP-1 restores the intestinal barrier function in DSS-induced colitic mice

**Figure 4:**
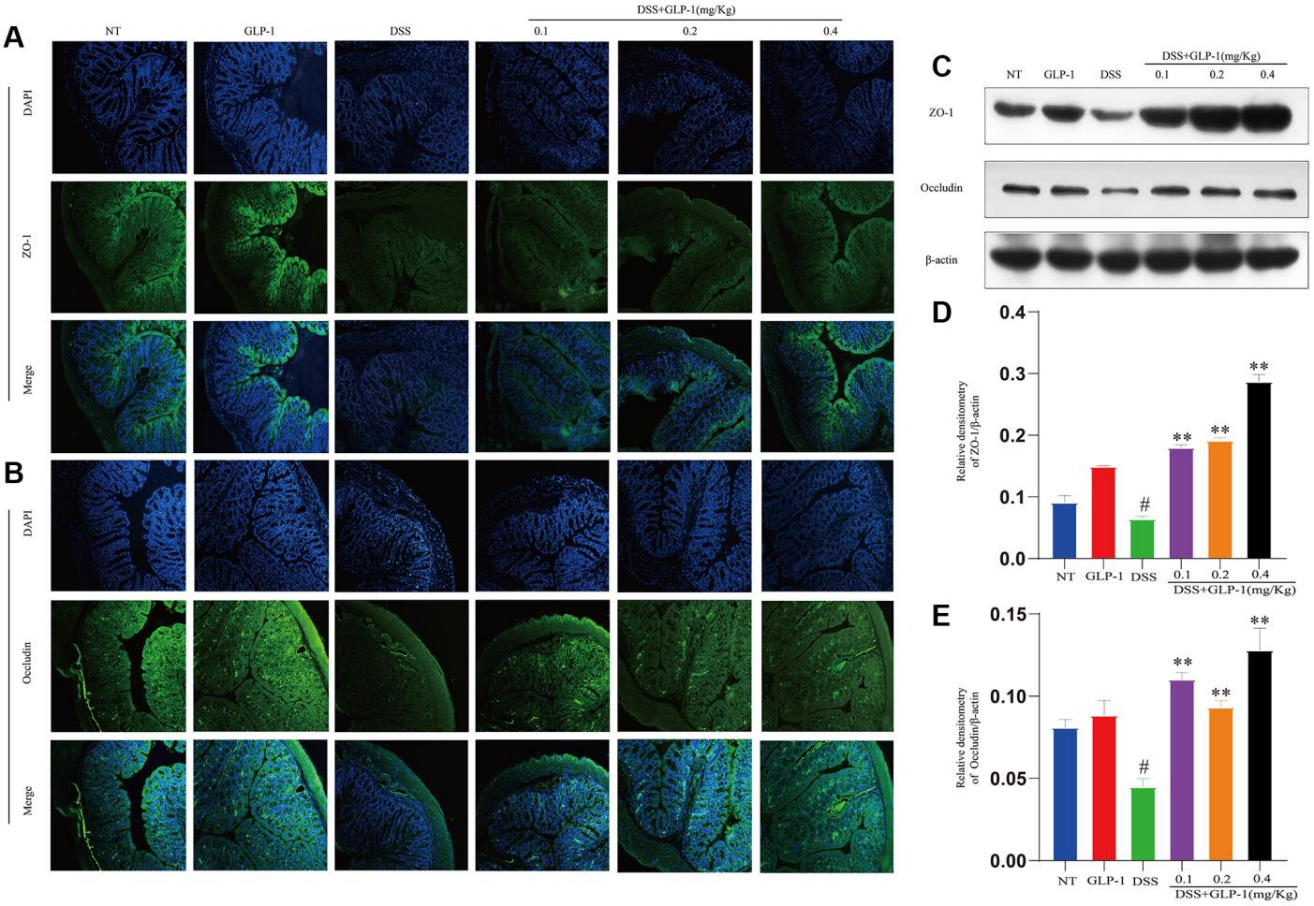
**GLP-1 restores the intestinal barrier function in DSS-induced colitic mice.** The protein levels of ZO-1 (**A**) and Occludin (**B**) were analyzed by immunofluorescence in the colon tissue. The protein expression of ZO-1 (**C**, **D**) and Occludin (**C**, **E**) were analyzed by western blotting. Data are presented as mean ± SD (*n* = 6 per group). For GLP-1-DSS vs. DSS group, ^*^p < 0.05 - ^**^p < 0.01 and for DSS vs. NT group, ^#^*p* < 0.05 were considered significant.

DSS-induced local and systemic proinflammatory mediators led to the disruption of the intestinal barrier. The normal structure and function of the intestinal barrier depend on the tight junction proteins. Therefore, we examined the expression level of two proteins, ZO-1 and Occludin, by western blot and immunohistochemistry. The expression of ZO-1 and Occludin was significantly downregulated in the DSS treated group but was restored with GLP-1 treatment (Figure 4).

### GLP-1 affects gut microbiota in DSS-induced colitic mice

**Figure 5:**
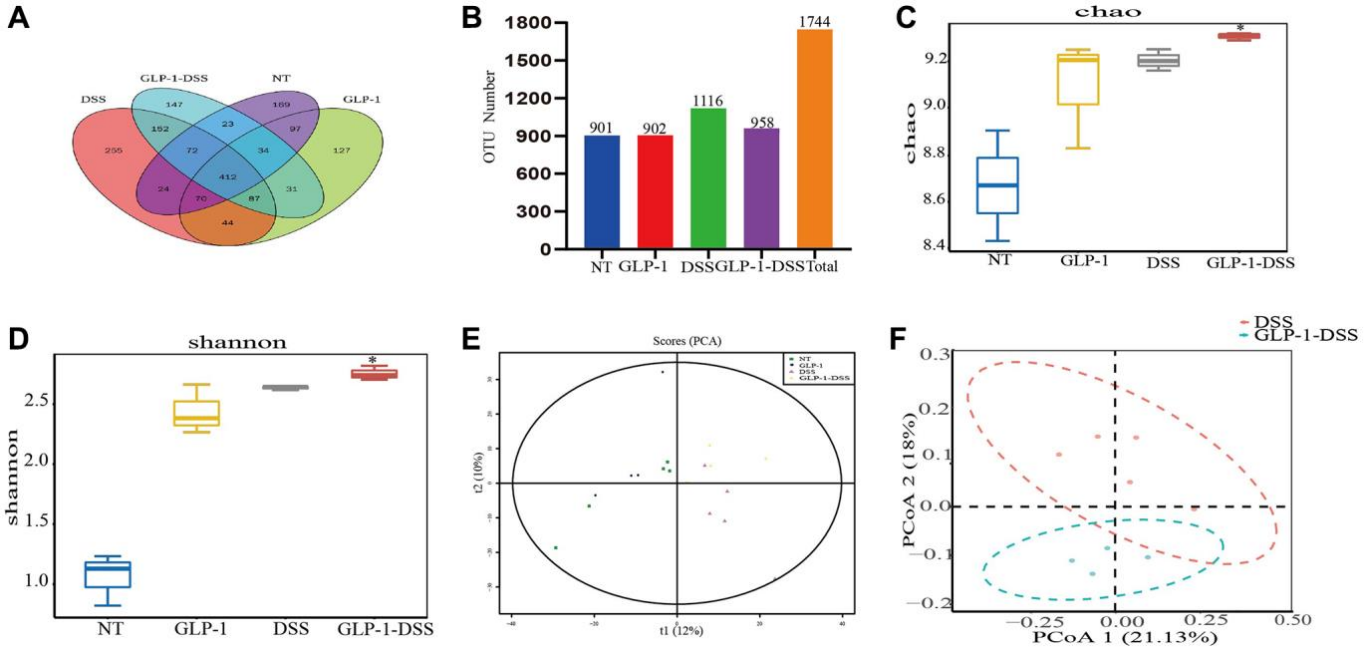
**The effect of GLP-1 on microbial diversity.** (**A**) OTU Venn chart. (**B**) The histogram of OTU number (Numbers show the total number of OTUs in each group). The α-diversity shows the community richness estimated with Chao 1 index (**C**) and Shannon index (**D**); the β diversity shows the diversity of bacteria in the cecum through PCA (**E**) and PCoA (**F**) analysis. For GLP-1-DSS vs. DSS group, ^*^p < 0.05 was considered significant.

Based on the 16S rDNA high-throughput sequencing, we started with 1521 OTUs to investigate the effect of GLP-1 on intestinal microflora in mice with colitis. Fecal samples from NT, GLP-1, DSS and DSS + GLP-1 (0.4 mg/Kg) groups were selected for microbial sequencing analysis. Mice given 0.4 mg/Kg GLP-1 showed better colon protection effect (increased colon length and decreased inflammatory cytokine secretion) than mice given 0.1 and 0.2 mg/Kg GLP-1 at low and medium doses, but no difference in weight loss. Therefore, we selected DSS-GLP-1 (0.4 mg/Kg) mice for sequencing analysis. The total OTU number was 1744 defined by 97% sequence similarity. The identity number of the respective OTU in the NT, GLP-1, DSS, and DSS-GLP-1 groups were 901, 902, 1116, and 958, respectively (Figure 5A, 5B).

### α- and β-diversity analysis

The α-diversity analysis was mainly related to the richness and diversity of the microbial communities. There was a sharp decline in the Chao 1 and Shannon indices in the DSS-induced mice model compared to the GLP-1 treated group (*p* < 0.05) (Figure 5C, 5D), indicating that GLP-1 treatment promotes the diversity of the bacterial community. The analysis of β-diversity by PCA and PCoA plots showed a remarkable difference in the species diversity between the control and the DSS-induced mice (Figure 5E, 5F). Interestingly, GLP-1 treatment enhanced the richness and diversity of the microbial community (Figure 5).

### Gut microbiome analysis

**Figure 6:**
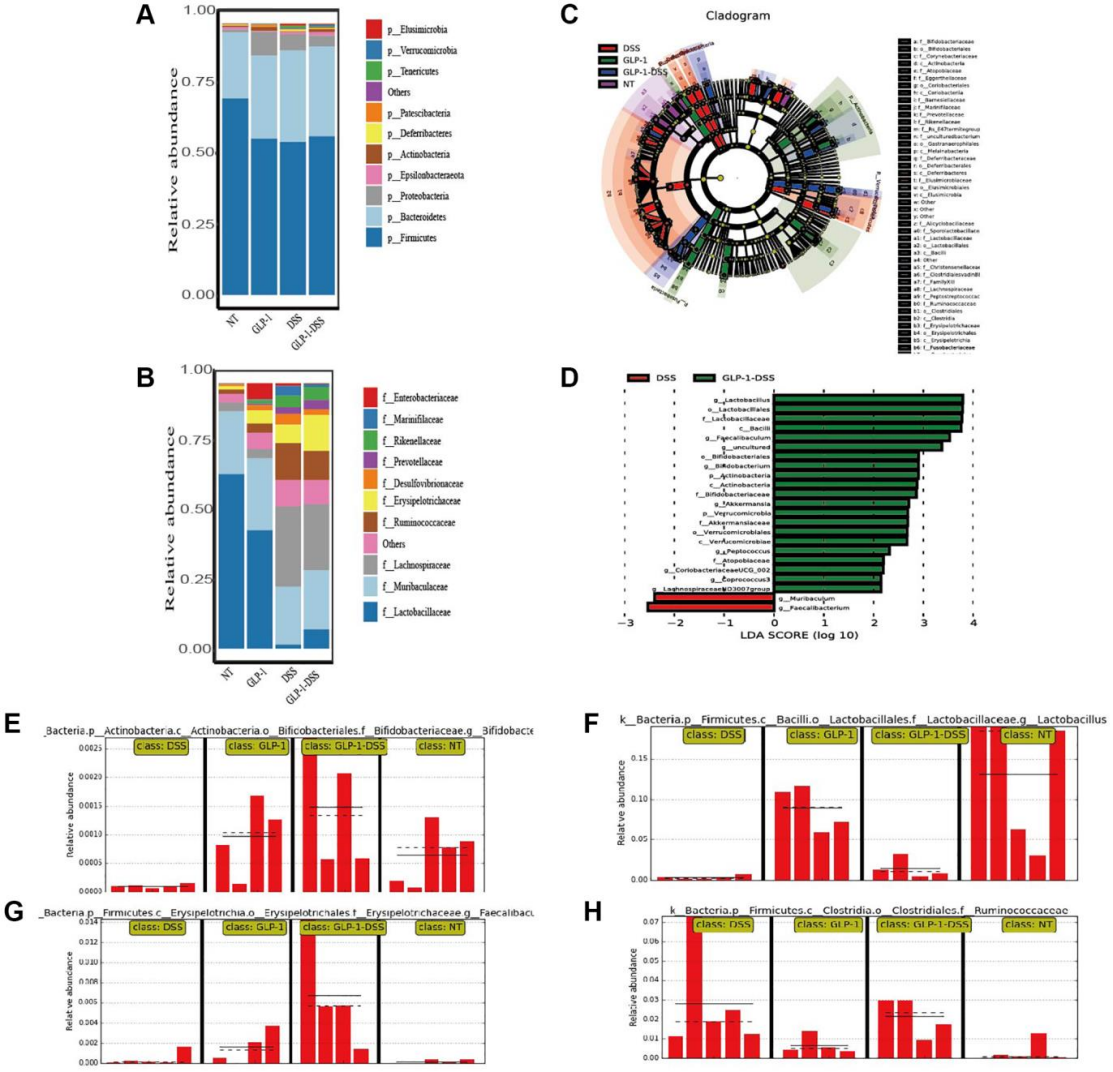
**The effect of GLP-1 on the microbial community of the gut.** (**A**) The top 10 phyla in colon samples from the four groups. (**B**) The top 10 families in colon samples from the four groups. (**C**) LEfSe cladogram. (**D**) LDA value distribution histogram. LDA score ≥2. Relative Bifidobacteriaceae (**E**), Lactobacillaceae (**F**), Faecalibaculum (**G**), and Ruminococcaceae. (**H**) Abundance in the colon based on the LefSe results.

To further investigate whether GLP-1 treatment influences the intestinal microflora, we tested the microbial composition among four groups of mice (Figure 6).

**Figure 7:**
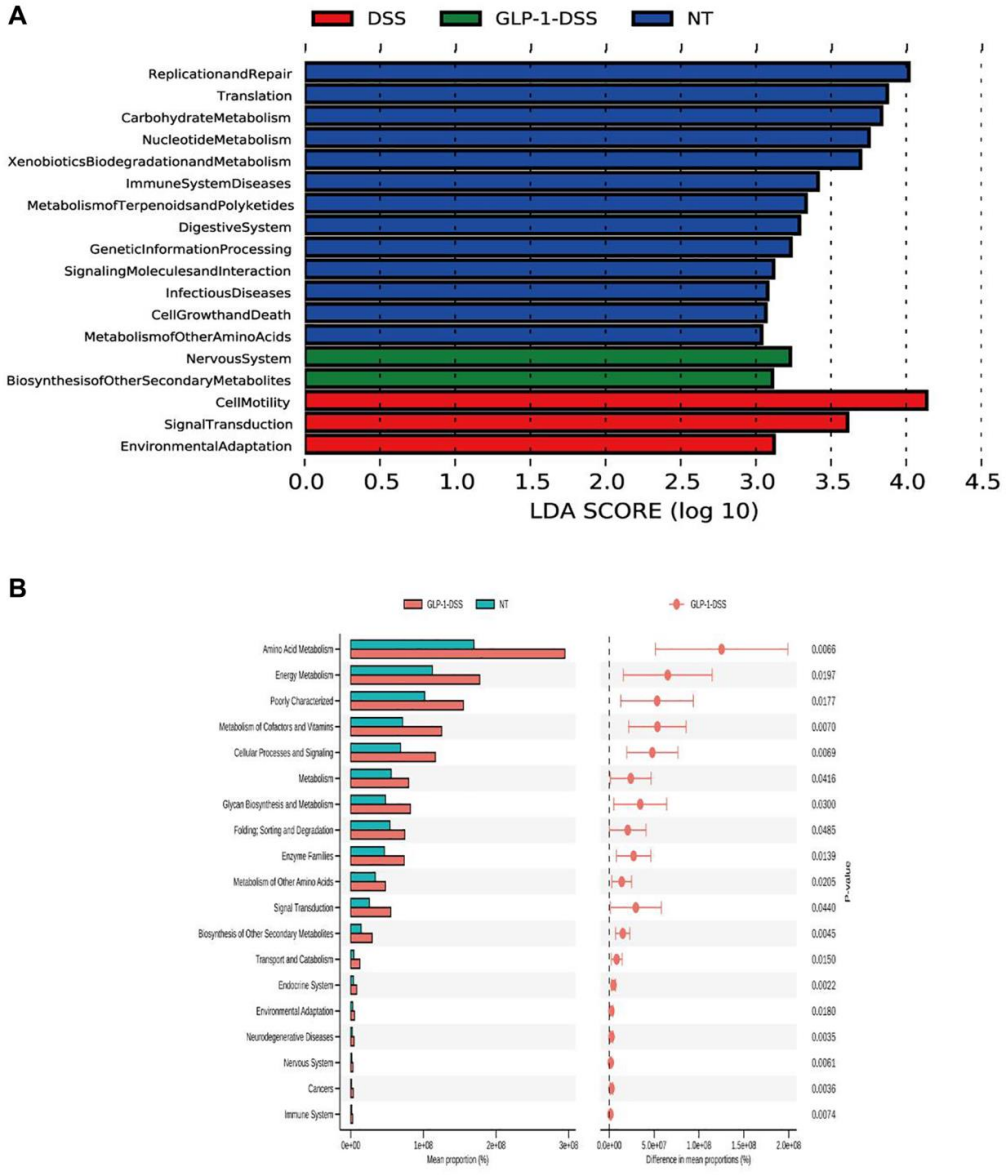
(**A**) Gene function prediction. (**B**) Differential KEGG pathways between DSS-GLP-1 and NT groups.

Analyses of relative microbial abundance at that phylum and family levels revealed that GLP-1 administration was linked to increase in the relative abundance of Firmicutes and Lactobacillaceae species in the GLP-1-DSS group relative to the DSS group (Figure 6A, 6B). The LEfSe (Line Discriminant Analysis (LDA) Effect Size) were next conducted to identify groups of bacteria that differed significantly among these four groups (Figure 6C, 6D). The relative abundance of Bifidobacteriaceae, Lactobacillaceae, Faecalibaculum, and Ruminococcaceae were higher after GLP-1 treatment than in DSS (*p* < 0.01) mice alone (Figure 6E–6H). GLP-1 re-established intestinal microbiota. We utilized COG and KEGG databases to analyze the functions of genes in the sequenced microbial genomes. We focused on proteins involved in signaling pathways (Figure 7). Nineteen aberrant enrichments KEGG pathways were displayed between DSS-GLP-1 and NT groups, including the microbial genes related to carbohydrate metabolism, nucleotide metabolism, lipid metabolism, metabolism of cofactors and vitamins, and amino acid metabolism.

### GLP-1 represses inflammatory response in LPS-induced RAW264.7 cells

**Figure 8:**
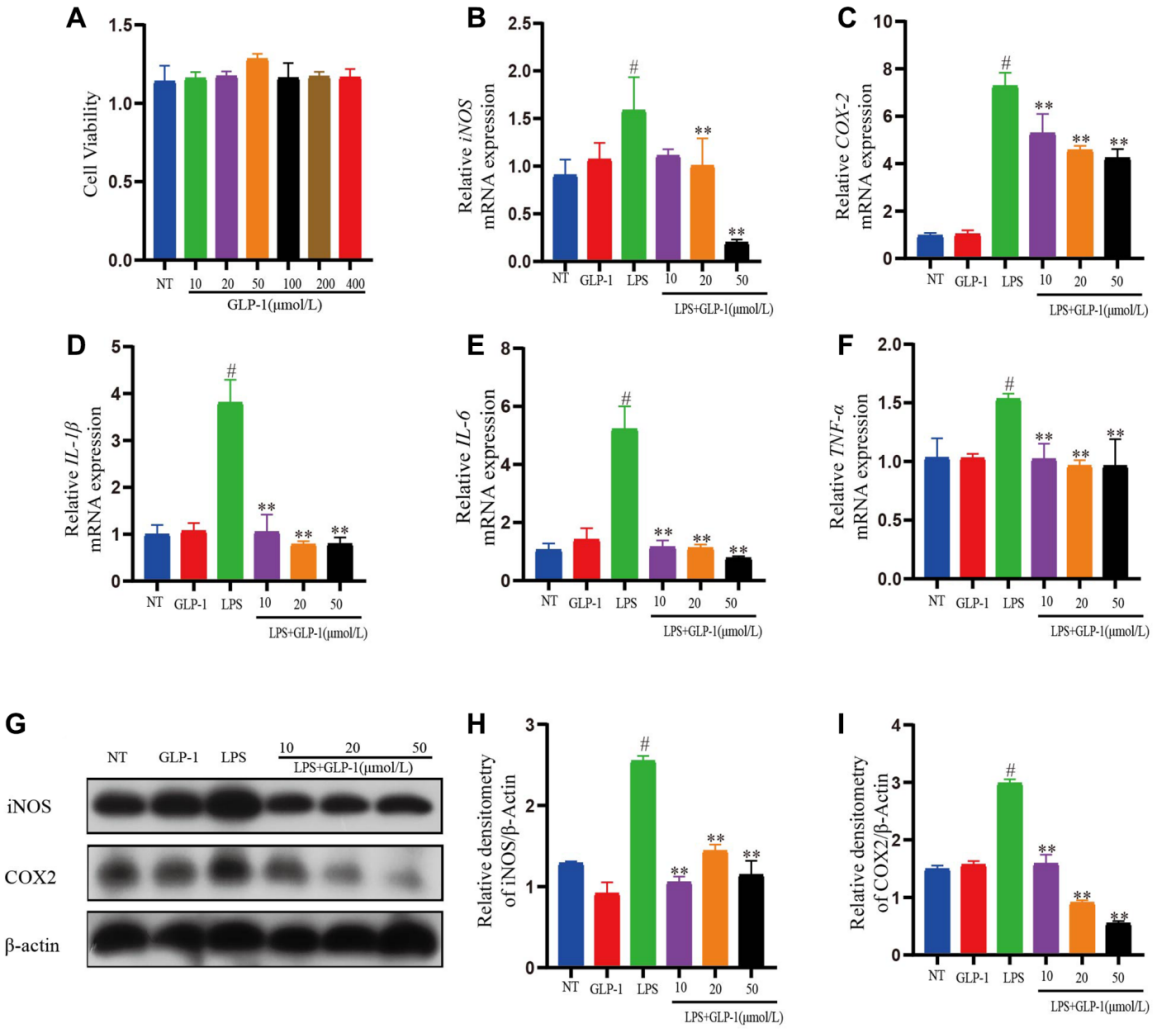
**GLP-1 represses inflammatory response in LPS-mediated RAW264.7 cells.** (**A**) Cell viability. The mRNA expression level of *iNOS* (**B**), *COX-2* (**C**), *IL-1β* (**D**), *IL-6* (**E**), and *TNF-α* (**F**) in LPS-induced RAW264.7 cells. The protein expression of iNOS (**G**, **H**) and COX-2 (**G**, **I**) was estimated by western blotting. The values shown here are the mean ± SD of three independent experiments. For LPS+GLP-1 induced RAW264.7 cells vs. the LPS group, ^*^p < 0.05 - ^**^p < 0.01 and for LPS-induced RAW264.7 cells vs. the NT group, ^#^*p* < 0.05 was considered significant.

To test the anti-inflammation activity of GLP-1 on RAW264.7 cells, we took GLP-1 solutions in different concentrations (10, 20, 50, 100, 200, 400 μmol/L) revealing it to be non-toxic (Figure 8A). LPS-induced RAW264.7 cells were incubated in three different GLP-1 solutions (10, 20, 50 μmol/L) for 4 h. The transcription of *IL-1β*, *IL-6*, and *TNF-α* was increased by LPS as expected, but GLP-1 treatment irrespective of their concentration (10, 20, 50 μmol/L) restored these levels (*p* < 0.01) (Figure 8D–8F). The result also showed that iNOS and COX-2 expression levels in LPS-induced RAW264.7 cells were significantly enhanced. Interestingly, GLP-1 inhibited the level of mRNA transcription and protein expression both of iNOS and COX-2 (*p* < 0.01) (Figure 8B, 8C and 8G–8I). The above results suggest that GLP-1 can suppress the inflammatory response in LPS-induced RAW264.7 cells.

### GLP-1 inhibits LPS-mediated phosphorylation of Akt/NF-κB p65 and MAPK in RAW264.7 cells

**Figure 9:**
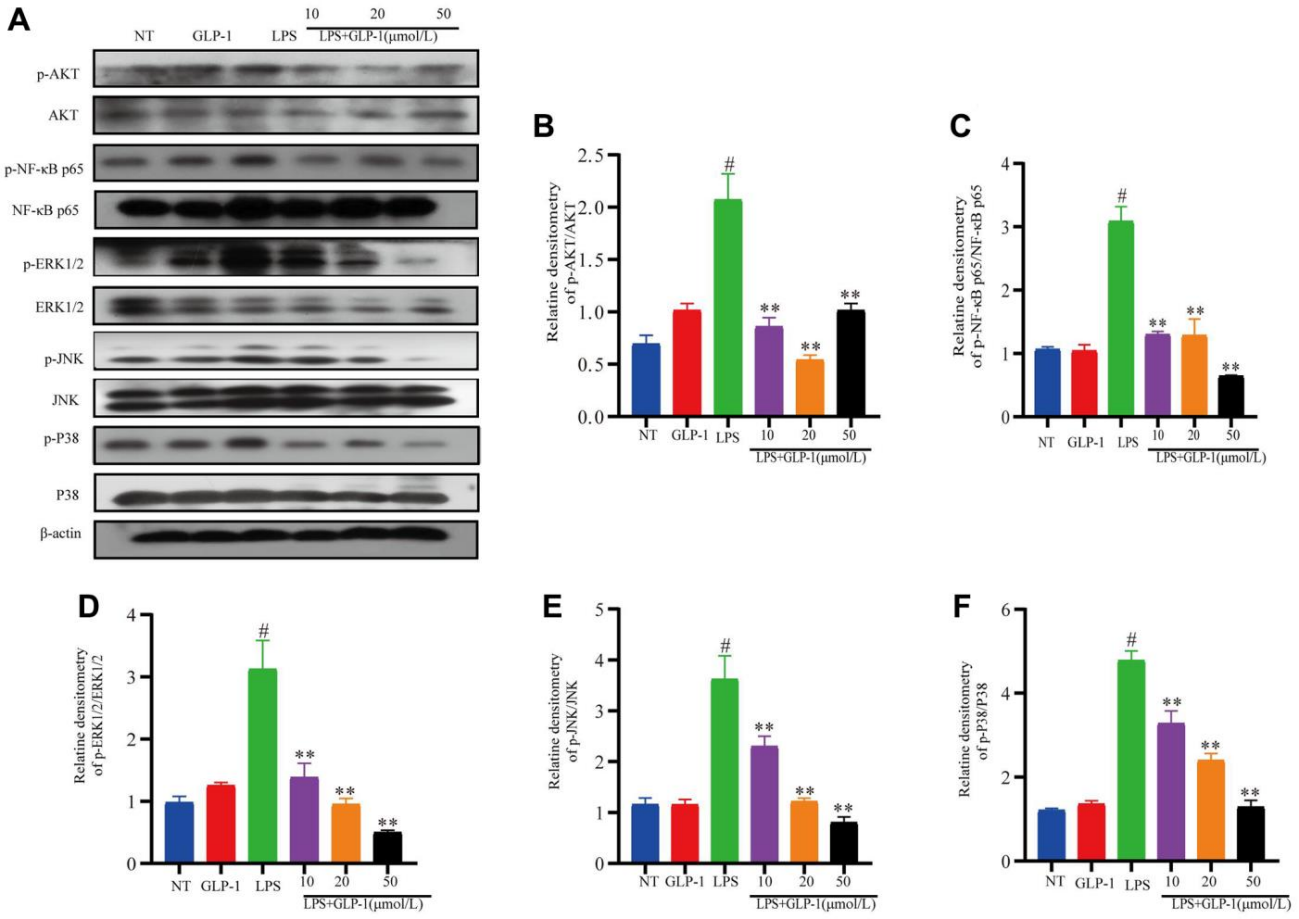
**GLP-1 inhibits the phosphorylation of AKT, NF-κB p65, ERK1/2, JNK and P38 in RAW264.7 cells.** The protein expression of p-AKT (**A**, **B**), p-NF-κB p65 (**A**, **C**), p-ERK1/2 (**A**, **D**), p-JNK (**A**, **E**) and p-P38 (**A**, **F**) was estimated by western blotting. The values shown here are the mean value ± SD of three independent experiments. For LPS+GLP-1 induced RAW264.7 cells vs. the LPS group, ^*^p < 0.05 - ^**^p < 0.01 and for LPS-induced RAW264.7 cells vs. the NT group, ^#^*p* < 0.05 was considered significant.

*In vivo* results have already shown that GLP-1 inhibits phosphorylation of AKT, NF-κB p65 and MAPK (this study). We also show that GLP-1 inhibits inflammatory pathways in the LPS-induced RAW264.7 cells. The level of phosphorylated NF-κB-p65 and AKT were also higher in these cells (Figure 9A–9C). Phosphorylation of ERK1/2, P38 and JNK increased in LPS-induced macrophages (Figure 9D–9F). Their levels were restored with GLP-1 treatment, similar to what we have shown in the mice model (this study).

## DISCUSSION

Around 6.8 million cases of UC have been reported worldwide, with a prevalence rate ranging from 79.5 to 84.3 per 100,000 people [2]. Current treatment of UC includes aminosalicylic acid, glucocorticoids, immunomodulators, and antimicrobial agents. But these drugs have severe side effects, and thus, the development of an effective therapy remains a top priority.

**Figure 10:**
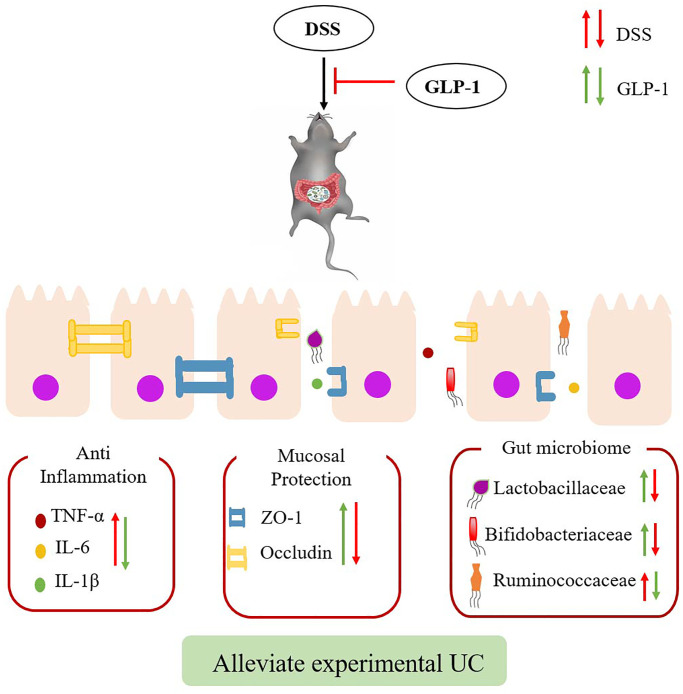
**Effects of GLP-1 on DSS-induced colitic mice.** The effect of GLP-1 (Green arrows) could alleviate DSS-induced (Red arrows) ulcerative colitis through reducing the secretion of inflammatory cytokines, increase the expression of tight junction proteins and regulating intestinal microbiota.

Apart from regulating blood glucose levels, GLP-1, the intestinal hormone, also has anti-inflammatory, anti-apoptotic, and antioxidative effects. Some studies have shown that GLP-1 alleviates macrophage-induced inflammation and relieves the symptoms of UC [18, 27, 28], but the underlying mechanism is still not completely understood. In this study, we have shown that intraperitoneal injection of GLP-1 reduces the effect of tissue injury in DSS-induced colitic mice by inhibiting intestinal inflammation, maintaining the intestinal barrier, and regulating intestinal microbiota. Apparently, GLP-1 has a protective effect on the LPS-induced RAW264.7 cell. Interestingly, GLP-1 alleviates inflammatory responses *in vivo* and *in vitro* by inhibiting phosphorylation of the AKT/NF-κB and MAPK signaling molecules. Our findings suggest that GLP-1 can be considered as a potential therapeutic candidate to prevent UC (Figure 10).

Proinflammatory cytokines, including IL-1β, IL-6, and TNF-α, can trigger the pathogenesis and promote the progressive development of UC [28]. The available clinical data suggest that anti-IL-6 and anti-TNF-α monoclonal antibodies have a certain ameliorative effect on patients with colitis [9]. It was proposed that the inflammation suppression role of GLP-1 relieves ulcerative colitis. In this study, GLP-1 inhibited the activity of MPO and affected the protein expressions of IL-1β, IL-6, and TNF-α in ulcerative colitic mice. In addition, GLP-1 significantly reduced DAI and improved colon length and histological manifestations of inflammation in mice. Macrophages secrete proinflammatory cytokines, participate in the innate immune response, damage tissues, and function in the development of UC [29]. GLP-1 also had anti-inflammatory effects in LPS induced RAW264.7 cells. These results suggest the protective effect of GLP-1 is related to its ability to regulate proinflammatory cytokines.

As already known, inflammation can induce colitis [30]. NF-ĸB, a family of transcription factors, contributes significantly to the inflammation process as it regulates the transcription of the inflammatory cytokines. A recent study has shown that NF-κB is a key player in the inflammatory pathway as it regulates the transcription of proinflammatory cytokines [31]. TNF-α overexpression can activate NF-κB leading to an aggravated inflammatory process [32]. Qiu et al. found that NF-κB can stimulate inflammatory cytokines and regulate gene expression in UC [33]. GLP-1R, liraglutide ameliorated inflammatory injury via activation of P38 MAPK/NF-κB signaling pathways [34]. In our study, both *in vivo* and *in vitro* experiments demonstrated that GLP-1 inhibits phosphorylation of NF-κB p65 and its upstream kinase AKT consistent with its anti-inflammatory role. We found that DSS stimulates the expression of p-NF-κB p65 and p-AKT, similar to previous findings [35–37]. These changes are markedly reduced in the GLP-1 treated mice.

The MAPK pathway is one of the common pathways for signal transduction in response to extracellular stimuli. MAPKs are comprised of protein serine/threonine kinases which include ERK1/2, JNK, and P38 [38, 39]. ERK1/2 and P38 are primary targets to curb inflammation, especially the P38 MAPK pathway, which participates in the regulation of LPS-induced inflammation response in macrophages [40]. The activation of ERK1/2 may be partly responsible for the elevated iNOS and COX-2 expression level induced by LPS in RAW 264.7 cells [41]. GLP-1R agonists activate the ERK1/2-signaling pathway in different types of cells [42]. Our results showed that GLP-1 dephosphorylates P38, ERK1/2 and JNK. GLP-1 treated LPS-induced RAW 264.7 cells showed repression of AKT/NF-κB and MAPK signaling pathways. GLP-1 analog liraglutide repairs structural damage in obesity-associated glomerulopathy and prevents sciatic nerve dysfunction [34] as it inhibits NF-κB and MAPK pathways. Therefore, we propose that the anti-inflammatory effect of GLP-1 is mediated through inhibition of AKT/NF-κB and MAPK signaling pathways by affecting the expressions of TNF-α, IL-6, and IL-1β. This study implies that targeting AKT/NF-κB and MAPK signaling pathways can be a way to ameliorate UC inflammatory change.

In DSS-induced colon inflammation, disruption of the mucosal barrier is the first event to trigger subsequent inflammatory cascade [10]. Liraglutide can minimize the increased permeability of the colon and facilitate mucosal healing after acute intestinal injury [8, 43]. These analyses are accordant with tight junction protein damage and local inflammation of intestinal epithelium in UC patients [44] and DSS-induced mice [31]. Our results suggest that GLP-1 can prevent mucosal barrier disruption and promote the production of tight junction proteins, Occludin and ZO-1, which inhibit the abnormal immune response in the intestinal mucosa and thus keep the intestinal mucosal barrier intact.

Disruption of the intestinal barrier and intestinal mucosal inflammation in UC is often coupled with the decrease in the number of bacterial species traditionally associated with beneficial effects on the host in the gut [45]. GLP-1 enhanced the richness and diversity of gut microbiota in DSS-induced colitic mice. Relative abundance of Lactobacillaceae, Bifidobacteriaceae, and Ruminococcaceae was most affected by DSS stimulation, but GLP-1 treatment helped Lactobacillaceae, Bifidobacteriaceae to survive and flourish. Both Lactobacillaceae and Bifidobacteriaceae can alleviate inflammatory bowel disease by modulating immune responses and altering the composition of the intestinal microbiota. Further LEfSe analysis showed that Lactobacillus and Bifidobacterium played an essential role in regulating intestinal microbiota diversity. Anaerobes like Lactobacillaceae and Bifidobacteriaceae can produce lactic acid and thus are associated with energy production in humans and other animals by increasing the levels of short-chain fatty acids (SCFAs) [46] in the gut.

A recent study has indicated a link between the composition of intestinal microbes and the risk factors involved in the development of UC, GLP-1 treatments increased Lactobacillaceae, Bifidobacteriaceae, and decreased Ruminococcaceae and Bacteroides. In clinical studies, Ruminococcaceae was enriched in the feces of patients with UC [47]. Thus, GLP-1 treatment improved the diversity of gut microbiota, increased the bacterial species traditionally associated with beneficial effects on the host (Lactobacillaceae and Bifidobacteriaceae), and decreased the bacterial species traditionally associated with harmful effects (Bacteroides and Ruminococcaceae). However, Ruminococcaceae usually acts as potential probiotics to ameliorate colitis, stressing more studies to clearly understand the effects of intestinal microflora in maintaining the intestinal epithelial layer integrity and the manifestation of UC.

Analyses using the COG and KEGG databases suggest that GLP-1 may participate in the pathways related to translation, immune system disorders, environmental adaptation, and cell motility. The growth of Bifidobacterium, suppresses the growth of potential pathogens, suppresses the expression of inflammatory factors, and regulates the immune system [48]. In our study, GLP-1 treatment increased carbohydrate metabolism, nucleotide metabolism, lipid metabolism, metabolism of cofactors and vitamins, and amino acid metabolism pathways, which may have some anti-inflammatory effects, and maintaining the energy and protein balance to improve intestinal inflammation and epithelial cell barrier [49].

In conclusion, this study focused on the positive and protective effects of GLP-1 in the DSS-induced colon inflammation in mice. GLP-1 can help reduce inflammation and membrane injury and regulate intestinal microbiota. Thus, GLP-1 can be considered as a promising therapeutic option to treat intestinal colitis. Finding a way to increase the biological half-life of GLP-1 or using GLP-1 analogs could be a step in the right direction to expand the treatment options for UC.
